# Phasing and structure of bestrophin-1: a case study in the use of heavy-atom cluster compounds with multi-subunit transmembrane proteins

**DOI:** 10.1107/S2059798315022524

**Published:** 2016-03-01

**Authors:** Veronica Kane Dickson

**Affiliations:** a Cambridge Institute for Medical Research, University of Cambridge, Hills Road, Cambridge CB2 0XY, England

**Keywords:** bestrophin, tantalum bromide, membrane-protein crystallography, detergents, antibodies

## Abstract

The purification and three-dimensional crystallization of membrane proteins are commonly affected by a cumulation of pathologies that are less prevalent in their soluble counterparts. This may include severe anisotropy, poor spot shape, poor to moderate-resolution diffraction, crystal twinning, translational pseudo-symmetry and poor uptake of heavy atoms for derivatization. Such challenges must be circumvented by adaptations in the approach to crystallization and/or phasing. Here, an example of a protein that exhibited all of the above-mentioned complications is presented. Bestrophin-1 is a eukaryotic calcium-activated chloride channel, the structure of which was recently determined in complex with monoclonal antibody fragments using SAD phasing with tantalum bromide clusters (Ta_6_Br_12_·Br_2_). Some of the obstacles to obtaining improved diffraction and phasing for this particular channel are discussed, as well as the approach and adaptations that were key to determining the structure.

## Abstract 2

The structure of a eukaryotic ion channel solved using tantalum bromide SAD phasing is discussed in the context of basic challenges common to membrane proteins.

## 1. Introduction   

Progress towards understanding the molecular mechanisms of membrane proteins *via* the elucidation of atomic resolution structures has lagged behind that of soluble proteins, largely because of obstacles to high-level heterologous expression of stable functional protein. Having overcome the initial hurdles and having achieved crystallization of the protein of interest, many membrane-protein crystallographers are then faced with further challenges including anisotropic diffraction and difficulty with heavy-atom incorporation. Several articles offer overviews of approaches to membrane-protein handling and crystallography and alternatives to traditional crystallization methods (Lacapère *et al.*, 2007 ▸; Sonoda *et al.*, 2011 ▸; Hammon *et al.*, 2009 ▸; Kang *et al.*, 2013 ▸; Baker, 2010 ▸; Morth *et al.*, 2006 ▸; Caffrey & Cherezov, 2009 ▸), whereas in this work we will discuss several aspects of one example: the calcium-activated chloride channel.

Anion permeability through the membrane bilayer is regulated in part by calcium-activated chloride channels (CaCCs) including bestrophin-1 (Sun *et al.*, 2002 ▸; Hartzell *et al.*, 2005 ▸, 2008 ▸). CaCCs are expressed in most eukaryotic cell types and are required for functions ranging from epithelial chloride secretion to neuronal and cardiac excitability to olfactory transduction (Hartzell *et al.*, 2005 ▸). Mutations in bestrophins lead to retinopathies owing to a dysregulation of chloride in the retinal pigment epithelium (Marquardt *et al.*, 1998 ▸; Petrukhin *et al.*, 1998 ▸; Davidson *et al.*, 2009 ▸). The channel has a high affinity for calcium (*K*_d_ of ∼150 n*M*) and has been proposed to have at least two domains of its primary sequence devoted to calcium binding, including a conserved stretch of highly acidic amino acids (Qu *et al.*, 2007 ▸; Xiao *et al.*, 2008 ▸; Kranjc *et al.*, 2009 ▸).

**Figure 1:**
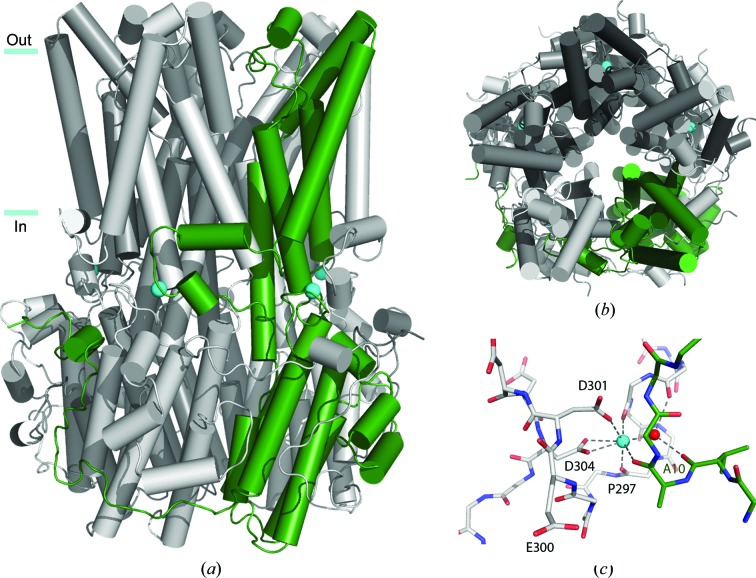
Structure of chicken bestrophin-1. The overall structure is shown as viewed (*a*) from the plane of the membrane and (*b*) from the outside of the cell. Each of the five subunits are identical and a single subunit is coloured green for clarity. Approximate boundaries of the membrane are indicated. Calcium ions are represented by cyan spheres. (*c*) Coordination in the Ca^2+^-clasp is shown including a coordinated water molecule (red sphere).

Bestrophin bears no primary sequence similarities to any other known family of anion channels, and the determination of its atomic structure revealed a unique fold (*DALI* server; Holm & Rosenström, 2010 ▸) featuring extensive domain-swapping between subunits. The bestrophin channel is a homopentamer comprising a central pore continuous with a large cytosolic region (Fig. 1 ▸). The pore is accessible extracellularly by a deep funnel-shaped entryway, narrowing to a hydrophobic gate region near the midpoint of the membrane and then widening to a positively charged intracellular cavity with only a small aperture to the cytosol. Anion binding is accomplished largely by interactions with helix dipoles, in favour of direct interactions with polar residues, which echoes a mode of stabilization seen in other anion channels and transporters. Intracellular calcium-binding sites are situated on the outside of the channel. Each subunit incorporates into the calcium-binding site domains its neighbouring subunit, termed the Ca^2+^-clasp, forming a belt-like structure of lateral helices at the cytosol–membrane interface. The coordination of the calcium ion is similar to that seen in EF-hand domains or the Ca^2+^-bowl, but the nature of communication between the Ca^2+^-clasp and the gate appears to be novel and requires further study (Kane Dickson *et al.*, 2014 ▸).

The initial purification and crystallization of bestrophin provided a good indication that structural studies would be achievable for this particular candidate. However, early diffraction images demonstrated marked anisotropy and poor spot shape, coupled with a very long unit cell. This crystal form also exhibited translational pseudo-symmetry. Here, we describe the path from the initial problematic diffraction to phasing and structure solution.

## 2. Candidate selection and purification   

### 2.1. Candidate selection   

Bestrophin exists in four isoforms in humans and most higher eukaryotes, denoted Best1, Best2, Best3 and Best4. The conservation of the primary sequence across all isoforms is very high for residues 1–390 (55% identity), but the remainder of the 600–700-amino-acid protein is not very conserved. Early screening involved cloning of both full-length and truncated forms of each isoform of the protein, where the truncated candidates were designed to end at the equivalent of human Best1 residue 398. This residue was selected in part owing to a report that calcium binding suffered if the protein was shorter than residues 1–380 (human Best1 numbering; Xiao *et al.*, 2008 ▸), and the sequence conservation was increasingly weak beyond residue 398. Protein that was truncated at this point was shown to be active using an assay of purified, reconstituted protein (Kane Dickson *et al.*, 2014 ▸). Of the 30 eukaryotic candidate orthologues expressed as a GFP fusion protein in HEK293 cells and screened by fluorescence size-exclusion chromatography (FSEC; Kawate & Gouaux, 2006 ▸), three were selected for further testing. Chicken Best1 residues 1–405 (GgBest1) was selected as one of the three top candidates. The protein demonstrated a monodisperse elution peak, as predicted by a sharp, symmetrical gel-filtration elution profile, in a range of detergents. The candidate was then moved from HEK293 cells to the larger-scale production system *Pichia pastoris*.

### 2.2. Purification and initial crystallization   

**Figure 2:**
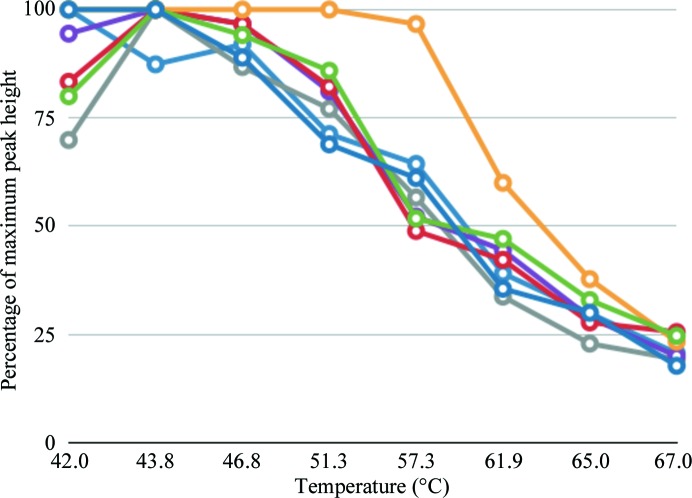
Effect of additives on the thermostability of GgBest1. Identical protein samples were treated at a range of temperatures between 42 and 67°C using a thermocycler and then assessed by size-exclusion chromatography monitored by tryptophan fluorescence. Traces are buffer control, blue; DMSO, light blue; 5 m*M* CaCl_2_, yellow; 50 m*M* GABA, green; 100 m*M* niflumic acid, red; 100 m*M* 5-nitro-2-(phenylpropylamino)benzoate (NPPB), lilac; 100 m*M* dihydro-4,4′-diisothiocyanostilbene-2,2′-disulfonic acid (DIDS), grey.

Purification was carried out using affinity purification *via* the anti-tubulin YL1/2 antibody and gel filtration using a Superdex 200 column. The purified channel demonstrated good characteristics as assessed by gel filtration and was functional as demonstrated by a flux assay (Kane Dickson *et al.*, 2014 ▸). A thermostability assay was carried out in the presence of a range of specific inhibitors and ions using analytical size-exclusion chromatography (SEC). To test thermostability, the protein sample was prepared (at a concentration of 1 mg ml^−1^), combined with the compound of interest and split into a control (20°C) and eight samples in a PCR reaction strip and then incubated over a temperature gradient between 42 and 67°C for 20 min using a thermocycler. Samples were then centrifuged at 20 800*g* for 30 min at 4°C and the supernatant was evaluated by the peak height of tryptophan fluorescence in SEC. Analytical SEC (and fluorescence SEC) has become a powerful diagnostic tool in assessing the stability of solubilized membrane proteins, wherein the shape and height of the elution peak is diagnostic of a monodisperse protein population. Gouaux and coworkers have published a similar thermostability protocol using fluorescence SEC (FSEC; Hattori *et al.*, 2012 ▸). Whereas using purified and untagged protein for this purpose has the advantage of reflecting the sample in the state that it will be used for crystallography or functional assays, using a GFP-tagged sample allows smaller sample sizes and could be carried out using solubilized lysate samples. Using purified protein for the purposes of this test, 20 µg of bestrophin was used per compound, which could be reduced tenfold if using FSEC. Alternatively, others have employed a fluorescent labelling method as described in Alexandrov *et al.* (2008 ▸) to assess thermostability. In this case, only Ca^2+^ was found to improve the stability of the purified protein (Fig. 2 ▸). As a result, 5 m*M* CaCl_2_ was added to the bestrophin samples used for crystallization in a high-calcium condition. The sample was routinely supplemented with 50 m*M* γ-aminobutyric acid (GABA) before use for crystallization trials. GABA was identified as an additive during crystal optimization *via* the screening of inhibitors and permeable species, as it has been reported to be a permeant species in glial cells (Lee *et al.*, 2010 ▸). GABA increased the number of well formed crystals in all crystal forms but was not required for crystallization.

The protein was solubilized in *n*-dodecyl-β-d-malto­pyranoside (DDM) and purified in DDM or another maltoside for crystallization (Kane Dickson *et al.*, 2014 ▸). Similar to the GFP-labelled protein in FSEC screening, the protein purified from *Pichia* also demonstrated stability over a range of detergents and formed crystals in several detergents tested. Initial diffraction patterns were collected from crystals formed in DDM, *n*-decyl-β-d-maltopyranoside (DM) and *n*-octyl-β-d-maltoside (OM). Diffraction ranged in quality but demonstrated severe anisotropy (over 110 Å^2^) that was not overcome by changes in the detergent or other crystal-optimization techniques. Crystals belonging to a rhombohedral space group (*R*32) were optimized and diffracted to 3.6 Å resolution in the best direction, but in addition to being severely anisotropic were also found to exhibit pseudo-translational symmetry.

### 2.3. Co-purification with an antibody fragment   

Following the observation of pathologies in the initial bestrophin-only crystals, a parallel approach was taken to find an alternate crystal form *via* the production of monoclonal antibodies. Selection of the antibody for use in crystallization involved eliminating those that bound to any unstructured protein (assayed by positive reactions to denaturing ELISA) and including a variety of populations that bound to structured regions. The Fab was prepared from purified IgG by papain cleavage and ion-exchange chromatography and was stored at −20°C. It was then quick-thawed and exchanged into a complementary purification buffer (identical buffer and salt composition but lacking detergent) immediately prior to binding. Affinity-purified GgBest1 was combined with purified Fab in a molar ratio of 1:1.2 and concentrated before application onto a final gel-filtration column. The sample was supplemented with 50 m*M* GABA and used directly for crystallization trials. Four Fabs were used for crystal trials and the final antibody selected generated two new crystal forms.

## 3. Crystallization and heavy-atom derivatization trials   

Bestrophin readily formed crystals in the detergents DDM, DM and CYMAL-6, among others. Optimized crystals which contained the GgBest1–Fab complex were grown by vapour diffusion either in CYMAL-6 or CYMAL-6 neopentyl glycol (CYMAL-6-NG) against a well solution consisting of 0–60 m*M* NaCl, 50 m*M* sodium acetate pH 4.0, 5% PEG 4000, 20% glycerol, generating crystals of the *P*2_1_ crystal form, or in DM against a well solution consisting of 120 m*M* NaCl, 50 m*M* Tris pH 8.5, 8.5% PEG 4000, 20% glycerol, generating crystals of the *C*2 form (Kane Dickson *et al.*, 2014 ▸). Both forms were grown by hanging-drop vapour diffusion using a 1:1 ratio of protein solution to well solution either in 100–600 nl drops (96-well format) or in 0.8–3.8 µl drops (24-well format). The crystals varied in size but were easily large enough for manipulation (over 50 µm). The *C*2 crystals required progressive stepwise soaking for dehydration in 25% PEG 4K. Both of these crystal forms suffered less anisotropic diffraction (of the order of 25 Å^2^) and had improved spot shape when compared with the bestrophin-only crystals. Translational pseudosymmetry was also absent, but the *P*2_1_ form was twinned when produced using protein purified in CYMAL-6 (twin fraction of up to 0.440; pseudomerohedral; operator *h*, −*k*, −*l*).

Neither of the crystal forms resulting from Fab-complex formation were successfully phased by molecular replacement (MR) using an Fab fragment or assembly as a search model. A post-mortem analysis of the failure of the MR trials revealed that one of the major issues derived from the placement of Fabs 2–5 in the helical density of the channel, which gave a higher score than placement in the β-sheet density of the Fabs. One Fab was not sufficient for phasing on its own as it comprised only 1/10 or 1/20 of the total mass of protein.

Crystals of both the *P*2_1_ and *C*2 forms were subjected to heavy-atom soaks in a wide range of derivatizing agents (Table 1 ▸; see also Morth *et al.*, 2006 ▸), some of which were more likely to bind than others given the pH ranges that are optimal for each compound (mostly pH 6–8) and the low pH of the *P*2_1_ crystal form (pH 4). Many of the soaked crystals showed diminished diffraction, but incorporation of the heavy atoms was universally poor as indicated by their anomalous signal. Co-crystallization was also attempted for many of the compounds, especially multivalent cations that may associate with a calcium-binding site, but the anomalous signal was not improved. Note that although band-shift assays (changes in electrophoretic mobility upon binding heavy atoms; see Boggon & Shapiro, 2000 ▸; Bergfors, 2009 ▸) may help in selecting candidates for binding of heavy atoms to soluble proteins, it is not usually possible to gain useful information in this way for many membrane proteins owing to their size and behaviour on native protein gels.

## 4. Phasing with tantalum bromide   

### 4.1. Derivatization with tantalum bromide   

Data were collected from crystals produced using selenomethionine-substituted protein and from crystals produced in sodium bromide (NaBr), but these data sets were not useful for phasing. As the stoichiometry was unknown (Johnson *et al.*, 2013 ▸) but was expected to be between four and six subunits per channel (with the mass of a channel–Fab complex therefore being of the order of 300–500 kDa), it was concluded that perhaps the difficulty in phasing with bromide or selenium was owing to the large number of relatively weak sites produced by these derivatives, and phasing may be made possible by using a large cluster compound that could bind at a single site per subunit or even per channel. The only heavy-atom cluster tested for this protein crystal was tantalum bromide (Ta_6_Br_12_·Br_2_), but several others are now commercially available, including the magic triangle (Beck *et al.*, 2009 ▸) and tungsten-cluster salts (Rudenko *et al.*, 2003 ▸), that may be useful in similar circumstances by the same rationale.

Tantalum bromide (TaBr) has many useful properties as a derivatizing agent for membrane proteins in addition to its more historical use for large soluble assemblies (Knäblein *et al.*, 1997 ▸; Banumathi *et al.*, 2003 ▸). Crucially, it is stable over a wide pH range (4–8). Although some literature provided with TaBr (Jena Bioscience; http://www.jenabioscience.com/images/69db037406/PK-103.pdf) previously suggested the preparation of a stabilization solution supplemented with the cluster, the most practical way to deliver it to a crystal grown by vapour diffusion is as a solid by way of introduction directly into the drop (April 2015 revision). The drop can then be observed over time and should turn green in colour as the particulate is dissolved. This is beneficial since the detergent concentration is not usually known with any accuracy, therefore preparing stabilizing solutions and cryoprotection solutions for the addition of heavy-atom soaking solutions is time-consuming and often leads to the destruction of several samples. It is possible that delivery by this method also aids in preservation of the crystal by a slow introduction of the compound into the crystal. A second or third introduction of solid can also be carried out. In the case of bestrophin, two additions and an incubation of up to 7 d led to the highest anomalous signal. When the tantalum bromide is incorporated the crystals are also visibly green. It was possible to introduce TaBr in this way for hanging drops as small as 150 nl + 150 nl. It was essential that the remaining solid cluster compound was stored under argon, and derivatization was most successful when the TaBr sample used was less than two months old.

### 4.2. Phasing with tantalum bromide   

The pitfalls of derivatization of GgBest1 by TaBr were that it led to a decrease in resolution and changed the unit cell sufficiently to make it non-isomorphous to native crystals. SAD phasing was carried out using a tantalum bromide-derived *P*2_1_ crystal that diffracted to a resolution of 4.4 Å. A large variation in unit-cell parameters was also noted when TaBr was used for phasing of the bacterial Complex I (Efremov *et al.*, 2010 ▸).

**Figure 3:**
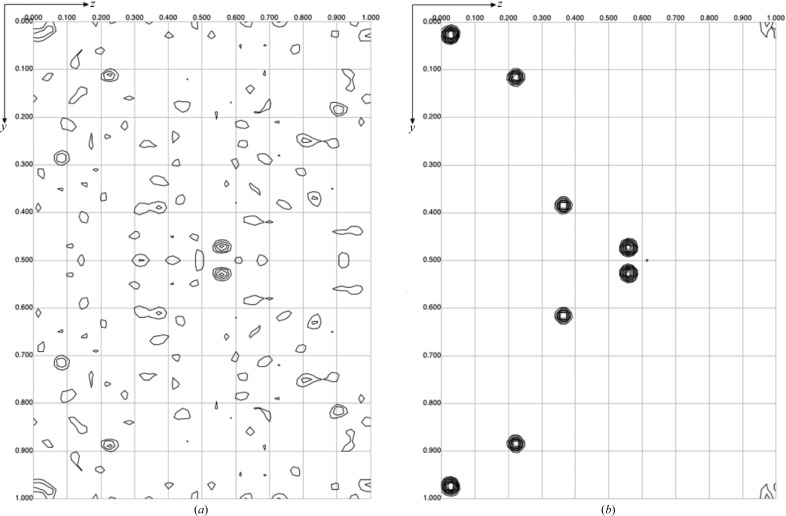
Anomalous difference Patterson map calculated in the resolution range 6–20 Å for a data set containing 25 TaBr molecules per unit cell (*a*) and the corresponding section of a synthetic map calculated from three unrefined sites (*b*). Maps are displayed using *MapSlicer*.

Even with a very high anomalous signal, phasing was not successful using fully automated methods for SAD or MAD. The Patterson maps generated from the successfully phased data set were complex, indicating either noise and/or a large number of sites (Fig. 3 ▸). The *SHELX* interface *via* *HKL*2*MAP* was used to identify candidate sites with strong cross-peaks and these were manually evaluated until three sites generated similar patterns in the synthetic Patterson maps to some of those seen in the experimental Patterson maps. Using these three sites, phasing was then possible *via* each automated method attempted, namely *SHARP*/*autoSHARP*, *Phaser EP* and *SHELX*. There were 25 cluster-binding sites found per channel (*i.e* five sites per bestrophin monomer; none in the Fabs), hence the complex maps observed. Occupancy was especially high (>0.8) for the top ten sites, which corresponded to two binding sites on the exterior of each bestrophin subunit near the calcium-bound Ca^2+^-clasp.

## 5. Structure solution   

**Figure 4:**
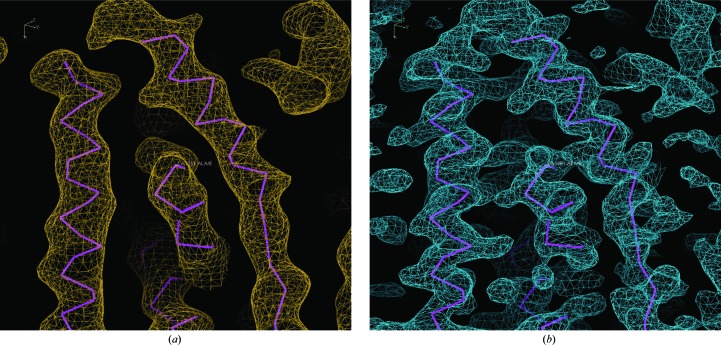
Comparison of electron-density maps at 4.4 Å resolution following phase extension and density modification without (*a*) or with (*b*) the application of fivefold NCS. The carbon backbone is shown in purple for reference only.

**Figure 5:**
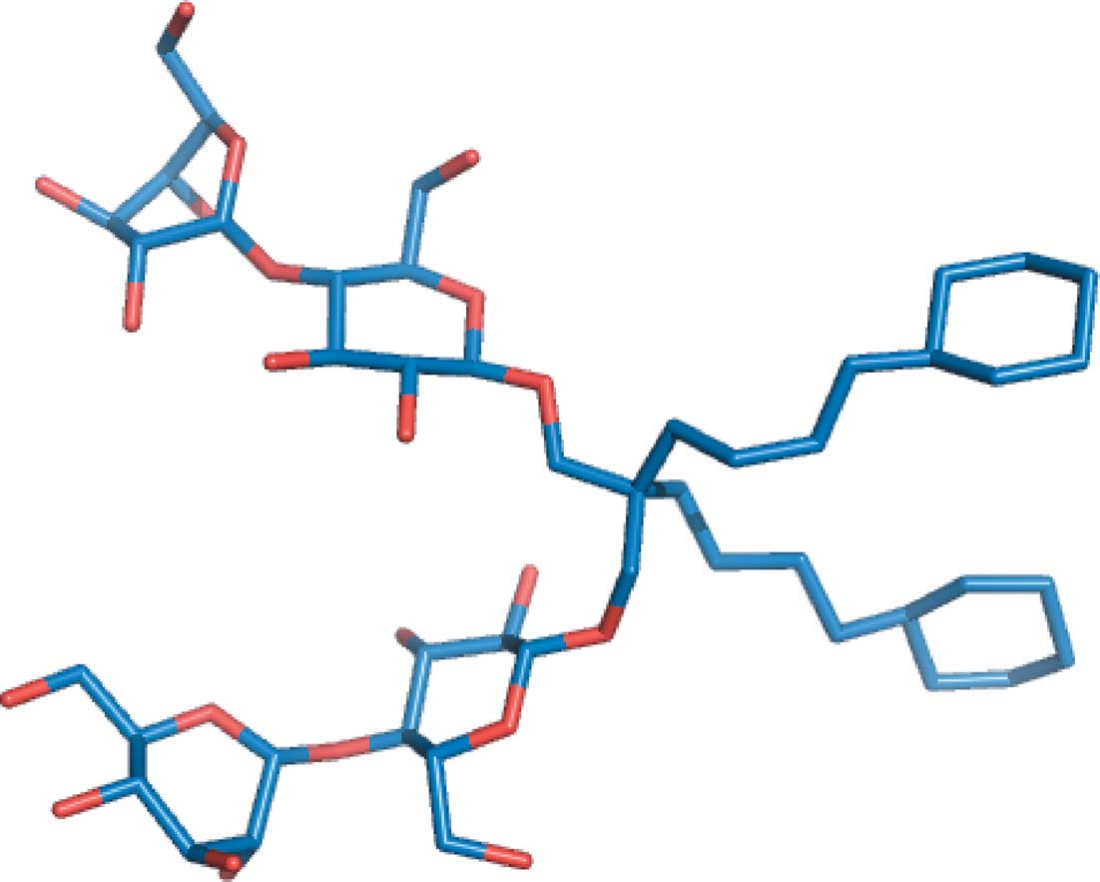
Three-dimensional structure of CYMAL-6 neopentyl glycol.

Phasing using TaBr was carried out using a *P*2_1_ crystal that diffracted to 4.4 Å resolution with phasing information to only 6 Å resolution. As these data were non-isomorphous to other data sets from crystals of the same form, structure building was stepwise and involved both the *C*2 and *P*2_1_ crystal forms. At an early stage it was not known whether the two forms would have the same structure owing to the vast difference in pH (8.5 for the *C*2 crystal and 4.0 for the *P*2_1_ crystal) and the possibility of a pH-dependent second calcium-binding site. Helical density and some side chains were clearly visible when fivefold noncrystallographic symmetry (NCS) was applied using operators generated by the positions of the TaBr clusters in combination with phase extension. Fig. 4 ▸ clearly demonstrates that it may not have been possible to build a useful model at this resolution had NCS not been applicable. An ideal helix model was built in the 4.4 Å resolution density, and the directionality of the helices was correctly estimated based on the appearance of the side chains. Fabs were manually placed owing to difficulties in placing them by MR. The ideal helix model in combination with the adjoining Fab was then successfully used as a MR search model for the *C*2 crystal form, where the best resolution available was 3.3 Å. The *C*2 crystal form had two channels per asymmetric unit and therefore had the benefit of tenfold NCS. Finally, the extended helix and loop model was placed back into the *P*2_1_ crystal form by MR, using a native data set collected with diffraction to at least 2.75 Å resolution. The register was then unambiguously placed using *ARP*/*wARP* and confirmed by the anomalous sulfur signals. Residues 2–367 were assigned with no breaks in density, including the extended loop region of the C-terminus (see the supplementary figure in Kane Dickson *et al.*, 2014 ▸). There was no discernible difference in the structure between the two crystal forms (r.m.s.d. = 0.2 Å) and no additional calcium-binding sites were observed. It is notable that the final improvement in diffraction of the *P*2_1_ crystal form was achieved by the use of a relatively new detergent class which has been reported to aid in the stability of membrane proteins. The detergent CYMAL-6-NG is comprised of two CYMAL-6 molecules bridged by a quaternary carbon that links their hydrophilic maltoside head groups to their hydrophobic alkyl chains (Fig. 5 ▸; see https://www.anatrace.com/Technical-Documentation/Technical-Documents/ProdSpec_Det_NG.aspx). Detergents of the ‘NG class’ have a much lower critical micellar concentratrion than their nonlinked counterparts and are thought to confer stability to solubilized membrane proteins by packing more tightly in the micelle (Chae *et al.*, 2010 ▸). An additional new class has recently been described as being stabilizing to membrane proteins and is characterized by a glyco-diosgenin (DGN) head group (Chae *et al.*, 2012 ▸). In the case of the GgBest1–Fab complex, CYMAL-6-NG allowed small improvements in the diffraction and reduction of the twin fraction when compared with the crystals obtained using CYMAL-6 (reduced from 0.225–0.440 to 0.017), which was key to achieving the best resolved density.

## 6. Discussion and conclusions   

The pursuit of the three-dimensional structures of membrane proteins by X-ray crystallography, although not without its challenges, has been rewarded in recent years and there are now over 500 unique membrane-protein structures (SBKB Membrane Proteins of Known Structure; White, 2015 ▸). In many cases improvements have been brought about by changes to the protein or its purification, mutation (Warne *et al.*, 2009 ▸), truncation (Hou *et al.*, 2012 ▸), the use of thermostable orthologues (Baradaran *et al.*, 2013 ▸) or cross-linking (Reyes *et al.*, 2009 ▸). However, even when an exceptionally stable sample is prepared, crystal packing between membrane-spanning regions in detergent-solubilized proteins is not generally observed. The presence of the detergent micelle around the purified membrane protein reduces the surface area available for forming crystal contacts relative to soluble proteins. The resulting crystal contacts can lead to anisotropy (and poor to moderate resolution) owing to the crystals growing better in one or two dimensions than in the third. When other issues such as poor uptake of heavy atoms or large unit cells compound these challenges, it can be useful to try phasing using large cluster compounds. These compounds have exceptional phasing power, are useful at resolutions as low as ∼8 Å and are likely to bind at fewer sites than smaller heavy atoms owing to their large diameters.

In the case of bestrophin, several incremental improvements were necessary to make the leap from initial diffraction to the final structure. Firstly, the screening and purification were optimized to find a stable and well expressed candidate. Secondly, multiple crystal forms and ultimately the addition of an antibody Fab improved the diffraction properties. Thirdly, tantalum bromide was a useful and successful derivatizing agent for phasing by SAD. Finally, changing the detergent in the crystals led to an improved proportion of crystals with good diffraction properties. In general, success in the pursuit of membrane-protein structures starts with a stable protein sample and benefits from the use of a multi-pronged strategy at each stage.

## Supplementary Material

PDB reference: bestrophin-1, 4rdq

## Figures and Tables

**Table 1:** Compounds used for heavy-atom soaking and co-crystallization

Compound	Anomalous scatterer
KAuCN_2_	Au
BaCl_2_	Ba
NaBr	Br
CdCl_2_	Cd
GdCl_3_	Gd
PCMB	Hg
Thimerosal	Hg
HgCl_2_	Hg
HoCl_3_	Ho
NaI	I
Ir_3_Cl_6_	Ir
LaN_3_O_9_	La
OsCl_3_	Os
Pt_3_Cl_6_	Pt
K_2_PtI_6_	Pt
RbCl	Rb
Sm(OAc)_3_	Sm
SrCl_2_	Sr
TlCl_3_	Tl
Na_3_[P(W_3_O_10_)_4_]	W
YbCl_3_	Yb
